# Estimation of air temperature and the mountain-mass effect in the Yellow River Basin using multi-source data

**DOI:** 10.1371/journal.pone.0258549

**Published:** 2021-10-21

**Authors:** Ziwu Pan, Jun Zhu, Junjie Liu, Jiangyan Gu, Zhenzhen Liu, Fen Qin, Yu Pan

**Affiliations:** 1 College of Geography and Environmental Science, Henan University, Kaifeng, China; 2 Key Laboratory of Geospatial Technology for the Middle and Lower Yellow River Regions (Henan University), Ministry of Education, Kaifeng, China; 3 Institute of Geographic Sciences and Natural Resources Research, Beijing, China; 4 Wuhan University, Wuhan, China; 5 Guizhou University, Guiyang, China; Universiti Teknologi Malaysia, MALAYSIA

## Abstract

Quantitative studies of the multiple factors influencing the mountain-mass effect, which causes higher temperatures in mountainous than non-mountainous regions, remain insufficient. This study estimated the air temperature in the Yellow River Basin, which spans three different elevation ranges, using multi-source data to address the uneven distribution of regional meteorological stations. The differences in mountain-mass effect for different geomorphic regions at the same altitude were then compared. The Manner–Kendall nonparametric test was used to analyse time series changes in temperature. Moreover, we employed the geographically weighted regression (GWR) model, with MODIS land-surface and air-temperature data, station-based meteorological data, vertical temperature gradients corresponding to the 2000–2015 period, and elevation data, to estimate the correlation between monthly mean surface temperature and air temperature in the Yellow River Basin. The following major results were obtained. (1) The GWR method and ground station-based observations enhanced the accuracy of air-temperature estimates with an error of only ± 0.74°C. (2) The estimated annual variations in the spatial distributions of 12-month average temperatures showed that the upper Tibetan Plateau is characterised by low annual air temperatures with a narrow spatial distribution, whereas north-eastern areas upstream of the Inner Mongolia Plateau are characterised by higher air temperatures. Changes in the average monthly air temperature were also high in the middle and lower reaches, with a narrow spatial distribution. (3) Considering the seasonal variation in the temperature lapse rate, the mountain-mass effect in the Yellow River Basin was very high. In the middle of each season, the variation of air temperature at a given altitude over the Tibetan Plateau was higher than that over the Loess Plateau and Jinji Mountain. The results of this study reveal the unique temperature characteristics of the Yellow River Basin according to its geomorphology. Furthermore, this research contributes to quantifying mountain-mass effects.

## Introduction

The mountain-mass effect, which was first reported by de Quervain [1] in the Alps, describes the higher altitude of temperature-related vertical belts (such as forest lines and snow lines) in mountainous areas than in non-mountainous areas or on isolated mountains. The distribution of this mountain-mass effect causes variations in geographic surface systems [2–5]. As a protruding heat island, mountains absorb solar radiation and convert it into long wave thermal energy, resulting in a much higher air temperature in mountain ranges than in the free atmosphere at the same altitude [4, 6]. The mountain-mass effect can be explained by the atmospheric heating process, where the ground is the main direct heat source for the atmosphere [7]. Due to the high altitude of mountains and the thin surrounding air, the ground in mountainous areas receives more solar radiation than that in non-mountainous areas; thus, the internal climate of a mountain range is relatively dry, with minimal precipitation, a reduced atmospheric weakening effect, and greater heat transfer to the atmosphere [5]. When air heated by the ground away from a mountain rises to the same altitude as a mountain range, its heat is greatly reduced. Furthermore, the mountain-mass effect is more obvious in the interior of mountain ranges than at the margin of mountainous areas; the larger the mountain, the higher the upper limit of plant growth and the higher the corresponding vertical vegetation zone boundary [8].

Additionally, the mountain-mass effect also influences the climate on the mountain itself [3]. Compared with lowland areas, the air pressure, temperature, and humidity are lower in mountainous regions; however, sunshine and radiation are more intense, and rainfall is observed at a certain height [9]. On a mountain hillside, the distribution of different climatic zones is somewhat similar to that from the equator to the bipolar climatic zone, with altitude regulating temperature at low latitudes [10]. Therefore, even at the equator, high mountains will experience snow all year round. Additionally, different hydrothermal conditions have different effects in the vertical direction, leading to various changes in the vegetation landscape [11]. Additionally, the mountain-mass effect is influenced by various factors [12, 13]. For example, larger mountains exhibit a more obvious mass effect [14].

Temperature data for the Yellow River Basin are scarce given its geographical characteristics and the fact that only a few meteorological observation stations exist in the area. Therefore, conducting in-depth and quantitative research on the mountain-mass effect in this region is particularly challenging [15–17]. Additionally, air-temperature data across the Yellow River Basin are predominantly obtained via a series of statistical analyses and spatial interpolation using observations from discrete meteorological stations. For example, Pan et al. [18] performed statistical analysis on observation data from 142 stations in the Yellow River Basin to study the interdecadal variations in temperature corresponding to the past 50 years. Their research revealed the temporal and spatial distribution of air temperature, as well as variations across the basin. Further, Huang et al. [19] used data from 52 stations in the Inner Mongolia region of the Yellow River Basin corresponding to the 1951–2012 period to study air-temperature variability and warming stagnation in the basin over the past 60 years. Ideally, these previous results could be used to determine the overall temporal variations in temperature across the river basin; however, they do not accurately reflect the detailed spatial distribution of air temperature [20, 21].

Presently, the most common method of determining air temperature at a given location is to use data from existing meteorological stations [12, 22]. Thus, the lack of stations in the Yellow River Basin, as well as its complex and diverse terrain, hinders analyses of the spatial variability of air temperature by this method [23, 24]). To obtain more accurate interpolation results, Hwang et al. [25] generated high-precision global temperature and precipitation interpolation values using longitude, latitude, and altitude as the variables, based on global meteorological observation data from 1950–2000 and 90-m-resolution Shuttle Radar Topography Mission (SRTM) topographic data. The development of thermal infrared remote sensing technology has facilitated the more efficient acquisition of air-temperature spatial data [10, 26, 27]. Air-temperature estimates derived from the geographically weighted regression (GWR) model based on MODIS land-surface temperature data reveal a good linear relationship between land-surface temperatures obtained via thermal infrared remote sensing and air temperature [28–30]. Furthermore, the primary advantage of the GWR model is that it identifies the spatial non-stationarity of the relationship between the response variable and explanatory variables [31–33].

Analysis of the mountain-mass effect and the variability between mountains at the same altitude is key to quantifying this effect. Therefore, this study uses MODIS data and existing air-temperature data to estimate air temperatures in the Yellow River Basin with high precision, then obtains the differences in air temperature between various terrain heights in the region to quantify the mountain-mass effect in the Yellow River Basin. Specifically, the aims of this study are: (1) to analyse the time series changes in air temperature and land-surface temperature using the Manner–Kendall (MK) nonparametric test [34, 35], considering the differences between air temperature and land-surface temperature based on the root mean square error (RMSE); (2) to estimate the average monthly air temperatures in the Yellow River Basin using the GWR analysis method with long-term MODIS land-surface and air-temperature data, as well as a digital elevation model (DEM), based on the spatial heterogeneity theory; and (3) to investigate the air-temperature distribution and mountain-mass effect, considering the unique topography and reanalysis data corresponding to the Yellow River Basin. Therefore, the results of this study provide basic data that reveal the unique geographical characteristics of the Yellow River Basin. Further, these findings can play an important role in the process of diversifying and quantifying mountain mass effects.

## Data and methods

### Study area

**Fig 1:**
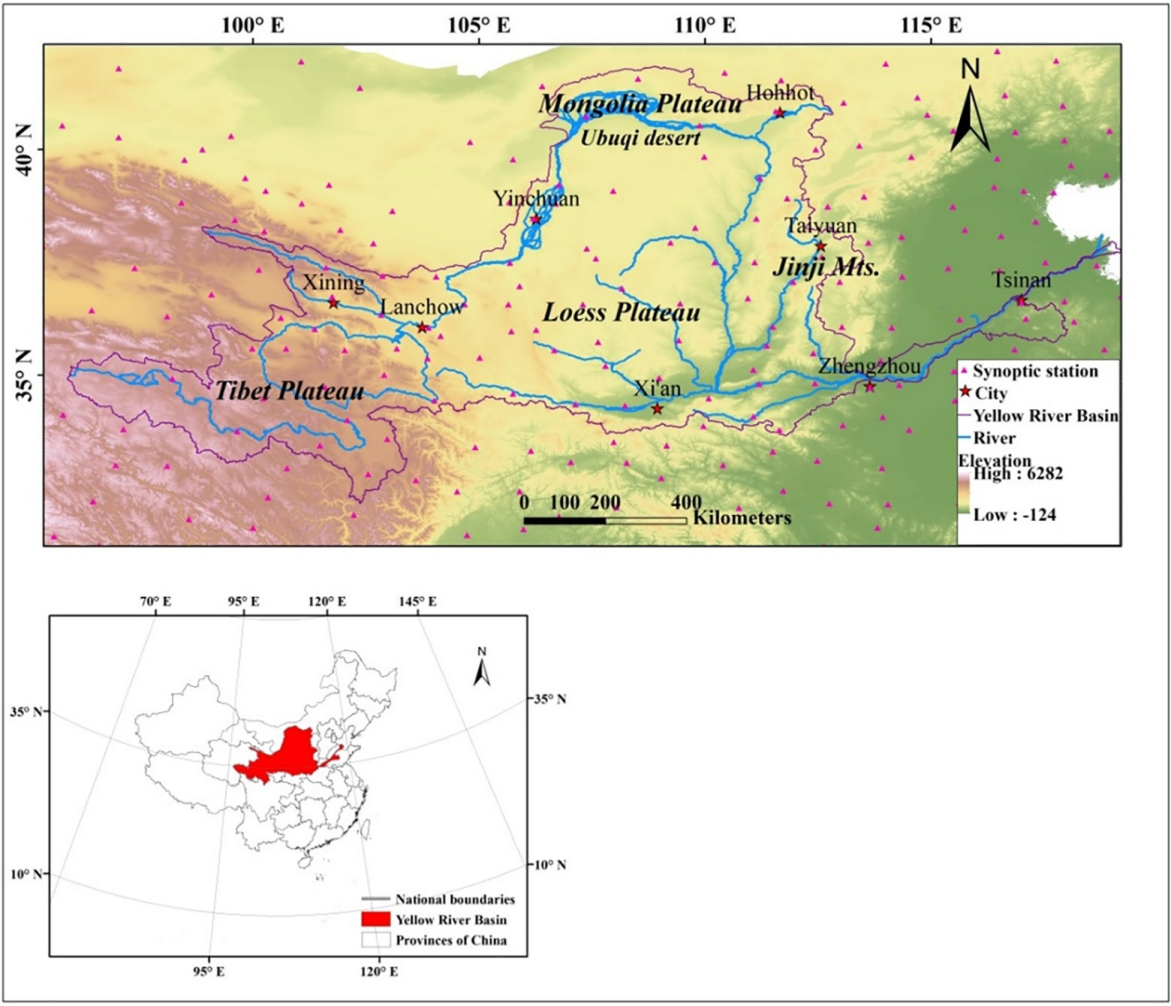
Study area and the distribution map of weather stations in this region.

The Yellow River Basin, which occupies a vast territory with highly varied topography, follows an east–west path across China (95°53′–119°05′ E, 32°10′–41°50′ N) (Fig 1). Its terrain is approximately divided into three zones: the western portion is located east of the Tibetan Plateau at an altitude of more than 3,000 m a.s.l, the central portion flows through the Loess and Mongolia Plateaus, at an elevation of 1,000–2,000 m a.s.l., and the eastern part flows through a plain with an altitude of 100 m a.s.l. The river basin encompasses plateau, middle temperate, and southern temperate climatic zones, and is ecologically fragile and sensitive. Additionally, meteorological stations are unevenly distributed throughout the basin; thus, comprehensive data on meteorological elements are scarce.

### Data

**Fig 2:**
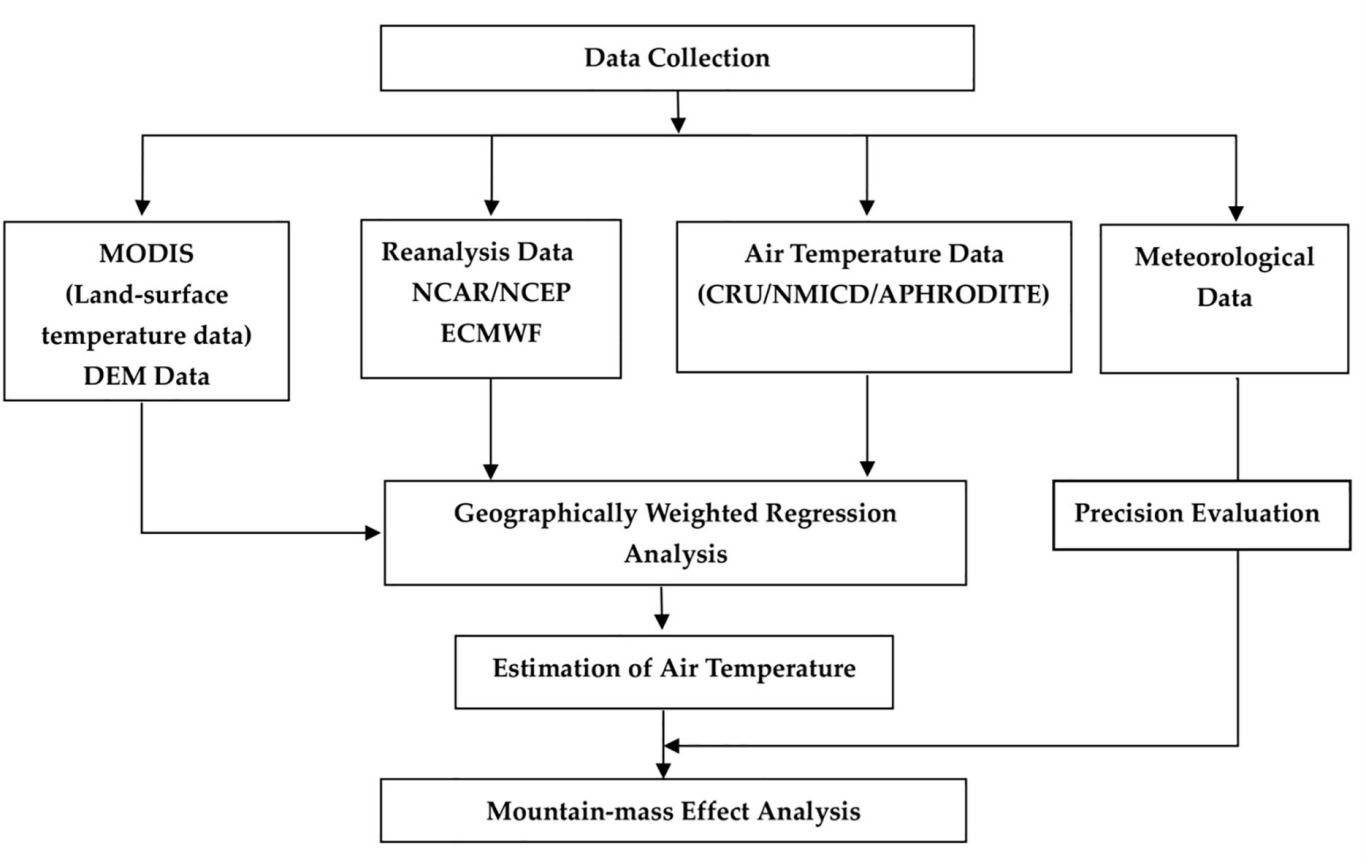
Flow chart depicting the mountain-mass effect analysis procedure.

In this study (Table 1 and Fig 2), MODIS Terra/Aqua global monthly mean surface-temperature/emissivity data for the 2000–2015 period, with a spatial resolution of 1 km, were used as land-surface temperature data. Terra/Aqua Monthly Land-Surface Temperature & Emissivity (MODLT1M/ MYDLT1M) data were also used. These data were obtained from the International Scientific & Technical Data Mirror Site (Computer Network Information Centre, Chinese Academy of Sciences). Owing to the presence of clouds, the MODIS surface-temperature data, specifically that corresponding to the middle of the Loess Plateau, contained missing values; therefore, the nearest neighbour method was employed to make local adjustments on the regularly and irregularly distributed data based on the structure of the input data, without requiring the user to enter data related to the search radius, sample count, or shape. The interpolation method used in this study tended toward the natural neighbouring method, which differs substantially from the traditional nearest neighbour interpolation method; however, the final results were relatively good. Missing values were replaced using the nearest neighbour method considering the elevation.

**Table 1:** Datasets used in this study.

Type	Name	Resolution
**Land surface temperature**	MODIS(MODLT1M/MYDLT1M)	1 km × 1 km
**Air temperature**	National Meteorological Information Centre Land Climate Data Monthly Value Data Set (NMICD)	1 km × 1 km
	Climatic Research Unit (CRU)	0.5° × 0.5°
	Asian Precipitation-Highly-Resolved Observational Data Integration Towards Evaluation (APHRODITE)	0.25° × 0.25°
**Reanalysis Data**	National Centre for Atmospheric Research/National Centres for Environmental Prediction (NCAR/NCEP)	2.5° × 2.5°
	European Centre for Medium Range Weather Forecasts (ECMWF)	0.25° × 0.25°
**DEM**	United States Geological Survey(USGS)	30 m
**Meteorological data**	National Meteorological Information Centre	---

Meteorological data were obtained from 225 stations in the study area during the 2000–2015 period (http://data.cma.cn/). The air-temperature data used in this study were average monthly temperature data corresponding to the 2000–2015 period, obtained from the National Meteorological Information Centre Land Climate Data Monthly Value Data Set (NMICD) (http://data.cma.gov.cn). The gridded Climatic Research Unit (CRU) time series dataset version 4.03 for the 2000–2015 period was also used to determine the daily mean temperature product (http://www.cru.uea.ac.uk/data/). Furthermore, Asian Precipitation-Highly-Resolved Observational Data Integration Towards Evaluation (APHRODITE) data, AphroTemp_V1808 for the 2000–2015 period were used as the daily mean 0.25 degree-gridded daily temperature product (http://aphrodite.st.hirosaki-u.ac.jp/index.html). Data from a 1-km resolution topographic DEM map of the Yellow River Basin were used as covariance values. The thin plate spline method was used for spatial interpolation to generate monthly grid data with a ground horizontal resolution of 1 km × 1 km.

Additionally, atmospheric temperature data were obtained from a reanalysis dataset provided by the National Centre for Atmospheric Research/National Centres for Environmental Prediction (NCAR/NCEP) (http://www.esrl.noaa.gov) and the European Centre for Medium Range Weather Forecasts (ECMWF) (https://www.ecmwf.int/). These data provide the monthly mean temperatures corresponding to 17 barospheres in the vertical direction. However, as the barometric pressure reaches the top of the troposphere at 250 hPa, and the rate of vertical temperature decline in the stratosphere differs from that in the troposphere, we select nine troposphere-pressure, 16-year (2000–2015) atmospheric monthly average temperature datasets corresponding to the following pressures according to the ideal atmospheric pressure and altitude table: 1,000, 925, 850, 700, 600, 500, 400, 300, and 250 hPa.

STRM DEM data with a spatial resolution of 30 m were downloaded from the USGS website (https://earthexplorer.usgs.gov/) and resampled within a grid unit of 1 km × 1 km to estimate air temperature using MODIS surface-temperature data.

### Methods

Average monthly land-surface temperature data from the MODIS Terra satellite corresponding to the 2000–2015 period were correlated with the average monthly air-temperature data from the meteorological stations. Further, average monthly Terra land-surface temperature data for day and night were averaged to obtain the monthly mean surface temperature in the Yellow River Basin from 2000 to 2015. A corresponding database was established for the following analyses:

1. The MK nonparametric test was used to analyse time series changes in temperature. The advantages of this method include the fact that the sample does not need to follow any specific distribution pattern, and interference from outliers is rare; therefore, the calculation is straightforward [31, 36–38].
2. The GWR method, which is a local statistical method that includes the spatial coordinates of variables during analysis and may provide a more appropriate basis for investigating spatially varying relationships, was used to explore the complex relationships between Ta, elevation, and land-surface temperature in the study area. The derived relationships were used to construct the Ta estimation model, whose performance was also compared to other estimation methods. Further, the GWR model is an extension of the conventional regression method and can be used to model spatially varying relationships [39]. In this model, the relationship between the dependent variable, Y_i_, and the explanatory variable, X_k,_ can be expressed as:

$$Y_{i}=\beta_{0}(u_{i},v_{i})+\sum_{k=1}^{p}\beta_{k}(u_{i},v_{i})x_{ik}+\varepsilon_{i}$$ (1)

where (u_i_, v_i_) represents the coordinates of the i^th^ location (e.g., latitude and longitude); β_0_(u_i_, v_i_) and β_k_(u_i_, v_i_) represent local coefficients estimated for the independent variable, x_k_, at point i; ε_i_ represents the regression residuals at location i; and i = 1,2,…,n, represents the number of spatial locations considered.

The regression coefficient of the independent variable in the formula was obtained according to the following formula:

$$\hat{\beta }(u_{i},v_{i})={\left(X^{T}(W(u_{i},v_{i})X\right)}^{-1}X^{T}W(u_{i},v_{i})Y$$ (2)

$$X=\left[\begin{array}{cccc} 1 & x_{11} & \cdots & x_{1k}\\ 1 & x_{21} & \cdots & x_{2k}\\ \vdots & \vdots & \ddots & \vdots \\ 1 & x_{n1} & \cdots & x_{nk} \end{array}\right],\;W(u_{i},v_{i})=W(i)=\left[\begin{array}{cccc} w_{i1} & 0 & \cdots & 0\\ 0 & w_{i2} & \cdots & 0\\ \vdots & \vdots & \ddots & \vdots \\ 0 & 0 & \cdots & w_{in} \end{array}\right]$$ (3)

$$\beta =\left[\begin{array}{cccc} \beta_{0}(u_{1},v_{1}) & \beta_{1}(u_{1},v_{1}) & \cdots & \beta_{k}(u_{1},v_{1})\\ \beta_{0}(u_{2},v_{2}) & \beta_{1}(u_{2},v_{2}) & \cdots & \beta_{k}(u_{2},v_{2})\\ \vdots & \vdots & \ddots & \vdots \\ \beta_{0}(u_{n},v_{n}) & \beta_{1}(u_{n},v_{n}) & \cdots & \beta_{k}(u_{n},v_{n}) \end{array}\right],\;Y=\left[\begin{array}{c} y_{1}\\ y_{2}\\ \vdots \\ y_{n} \end{array}\right]$$ (4)

where X and Y are matrices composed of explanatory variables and dependent variables, respectively; $\hat{\beta }$ represents an estimate of β; and W(u_i_, v_i_) = diag(w_i1_, w_i2_,…,w_in_) is a square matrix, whose diagonal element, w_ij_, denotes the geographical weighting of the surrounding observed point, j, for the specific point, i. This weight matrix, W(u_i_, v_i_), can be constructed using the distance, d_ij_, and the Gaussian distance decay-based functions.

The Gaussian and bi-square kernel functions are two common types of kernel functions that are part of the GWR model. The adaptive Gaussian kernel function was expressed as follows:

$$w_{ij}=\exp\left(-{\left(\frac{d_{ij}}{b}\right)}^{2}\right)$$ (5)

where d_ij_ represents the distance between the sites, i and j, and *b* represents an adaptive bandwidth size defined as the nearest neighbour distance. The adaptive bi-square kernel function was used to describe the spatial dependence of the data.


$$w_{ij}=\left\{ \begin{array}{l} {\left[1-{\left(\frac{d_{ij}}{b}\right)}^{2}\right]}^{2}\;d_{ij}\leq b\\ \;0\;d_{ij}>b \end{array}\right.$$ (6)

Further, it was necessary to carefully select an appropriate bandwidth, which can benefit from employing a measure of how well the model fits the data. Thus, an interval search was used to determine the optimal bandwidth of the adaptive bi-square kernel function, and the fitting degree and performance of the model were evaluated using the coefficient of determination (R^2^), adjusted R^2^, local R^2^, RMSE, and the square root of the standardised residual sum of squares (Sigma).

Additionally, the GWR method was used to estimate the regression equations for MODIS air temperature and land-surface temperature, as well as the altitude at each given location in the study area (Eq 7). It was also used to generate the land-surface temperature coefficient, altitude coefficient, and constant term coefficient, i.e., the regression model for temperature estimation was established using the GWR method.


$${Ta}_{i}(u)=\beta_{0i}(u)+\beta_{1i}(u)Ts_{i}(u)+\beta_{2i}(u)h_{i}(u)$$ (7)

Here, Ta represents air temperature; Ts represents the MODIS land-surface temperature; h represents the altitude; u represents a given spatial position; i = 1…n, represents the number of spatial positions considered; and β represents the corresponding coefficient terms.

In addition to using the GWR method to generate the land-surface temperature coefficient, air-temperature surface coefficient, altitude coefficient, and constant term coefficient, it was also used to establish the regression model employed to estimate the monthly mean air temperature in the Yellow River Basin. Even though the NMICD-based Ta data for the basin has a linear relationship with land-surface temperature, some spatial variability was still observed, which was estimated by introducing the altitude factor into the GWR regression analysis method. Further, to evaluate the accuracy of the model estimates, 225 meteorological stations in the study area were used as verification points.

3 The high-precision GWR model was also used to estimate the average monthly air temperatures in the basin for a period of 12 months. In this study, Ta at sea level was determined as described in a previous study [40]. Then, the average monthly air temperatures at altitudes of 5,000 m and 5,500 m in the Tibetan Plateau were determined to compare the differences between geomorphic regions in the basin:

$$Ta_{h}=Ta+(elevation‐h)\times \gamma$$ (8)

Here, h represents the specified altitude (5,000 m and 5,500 m); Tah and Ta are the simulated air temperature and actual temperature at the specified altitude, h, respectively; and γ represents the temperature lapse rate. Jiang et al. [41] used data from Chinese national meteorological stations to calculate the regional-scale lapse rates for the seasonal mean Ta based on a multiple regression method (Table 2).

**Table 2:** Lapse rates for mean air temperature at the regional scale (°C/100 m).

Area	Spring	Summer	Autumn	Winter
**Tibetan Plateau**	0.63	0.59	0.57	0.56
**Loess Plateau**	0.56	0.62	0.48	0.36
**Mongolia Plateau**	0.63	0.52	0.46	0.43
**Jinji Mountain**	0.54	0.57	0.49	0.52

4 In this study, we focused on analysing the characteristics and causes of tropospheric temperature gradient changes over the main parts of the Loess Plateau, Mongolia Plateau, Jinji Mountain, and Tibetan Plateau. The formula for determining the vertical temperature gradient was as follows:

$$\gamma_{T}=‐\Delta T/\Delta P$$ (9)

where γ_T_ represents the vertical temperature gradient (unit: K·hPa^-1^); ΔT = T_*k+1*_−T_*k*_ (k represents the level); ΔP represents the absolute value of the air pressure difference between two adjacent isobaric surfaces; and γ_T_ > 0 K·hPa^-1^ indicates that the temperature decreases as the altitude increases. Conversely, γ_T_ <0 K·hPa^-1^ indicates that temperature increases with height of the inversion layer and γ_T_ = 0 K·hPa^-1^ indicates that the temperature does not change with altitude. Further, the higher the γ_T_ value, the faster the temperature decreases with altitude.

## Results

### Data analysis results

**Fig 3:**
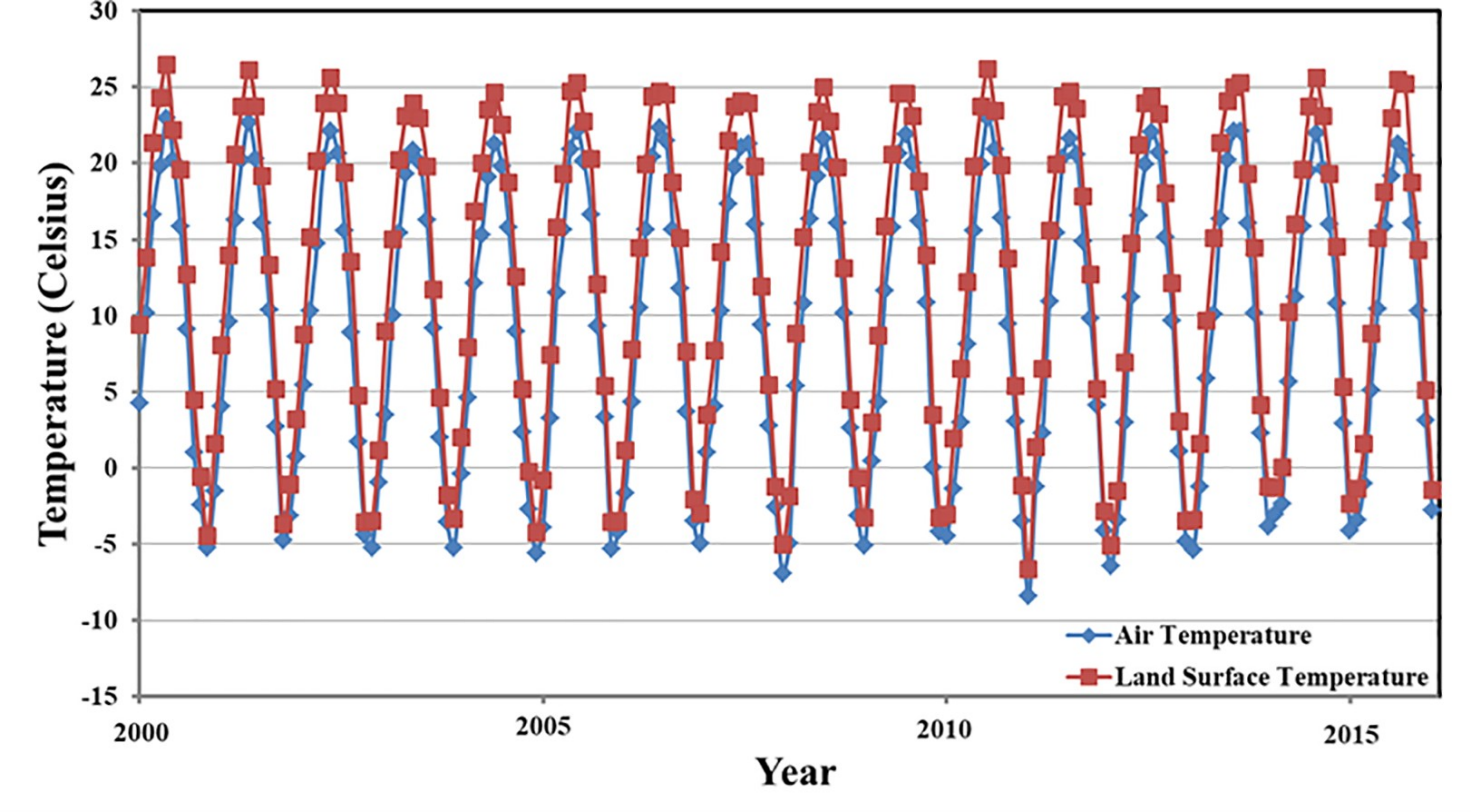
Temporal variations in monthly mean air temperature and land-surface temperature based on data collected at meteorological stations in the Yellow River Basin.

**Fig 4:**
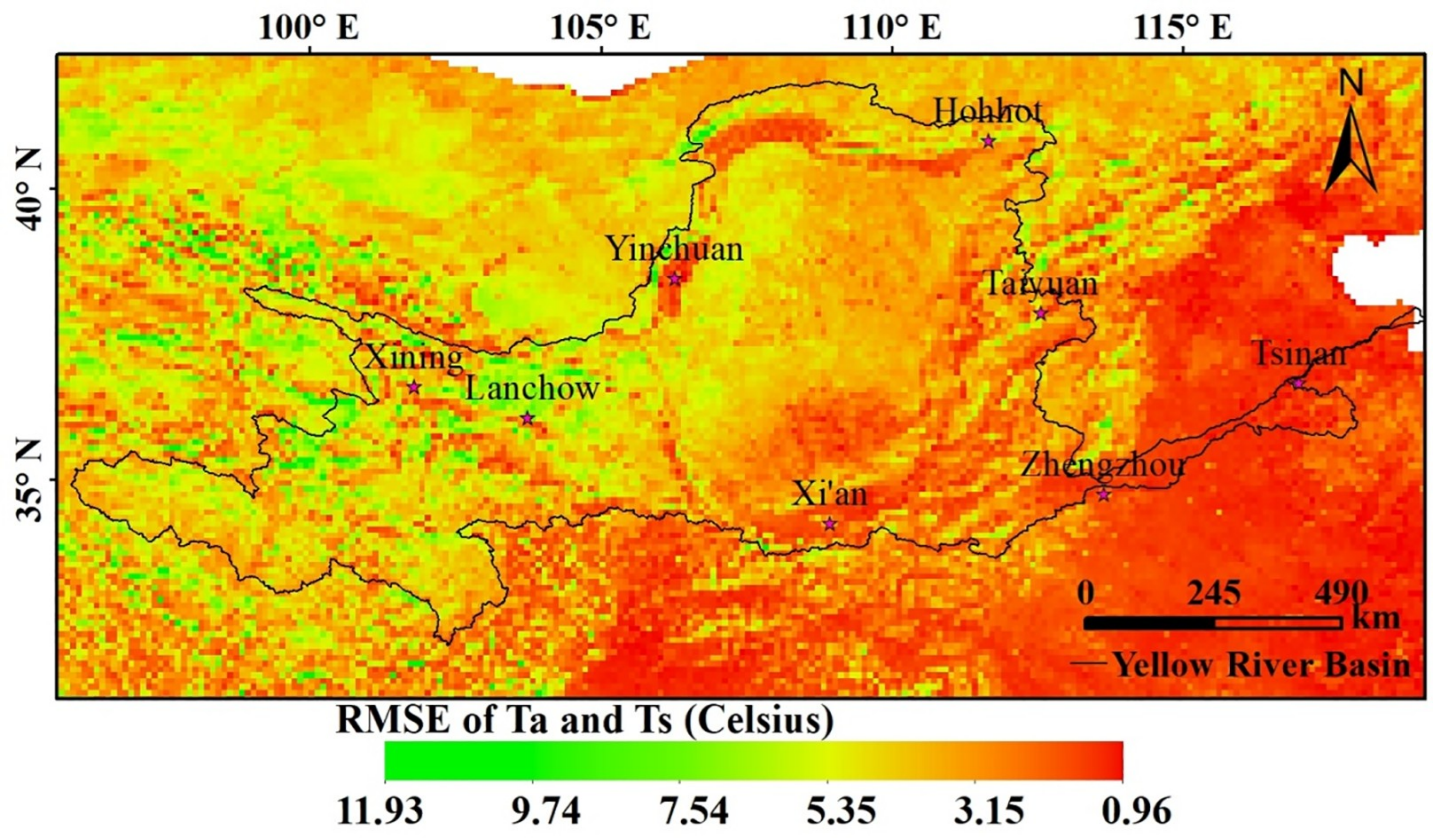
Spatial distribution of the Root Mean Square Error (RMSE) of air-temperature data (Ta) and surface-temperature data for the Yellow River Basin.

We compared different air-temperature products, including CRU [42], APHRODITE [43–48], and NMICD, to determine the most suitable product for this study. Even though CRU data and APHRODITE data had high *R*^*2*^ values (0.95–0.97 and 0.92–0.98, respectively), they also exhibited high RMSE values (1.02–1.26 and 0.99–1.66, respectively). Therefore, considering the resolution of the three different data products as well as their estimation accuracy results (Table 3), NMICD data were used in this study. The time series analysis performed using the MK nonparametric test indicated that the average monthly MODIS land-surface temperature was generally consistent with the change in NMICD-based average monthly Ta (Fig 3). The RMSE values corresponding to the central month of each season were statistically analysed (Table 4). RMSE values in the range of 48–82% indicated a temperature error of less than 4°C, whereas values in the range of approximately 2–19% indicated an absolute error of more than 6°C, which was primarily distributed in the upper reaches of the Tibetan Plateau, the eastern and western parts of the Loess Plateau, and in the middle part of the Inner Mongolia Plateau (Fig 4).

**Table 3:** Root Mean Square Error (RMSE) and coefficient of determination (R2) for different temperature products.

Month	NMICD		APHRO		CRU	
	R2	RMSE (°C)	R2	RMSE (°C)	R2	RMSE (°C)
**Jan.**	0.98	0.76	0.95	1.30	0.95	1.26
**Feb.**	0.96	0.94	0.92	1.49	0.96	1.02
**Mar.**	0.96	0.97	0.92	1.66	0.96	1.13
**Apr.**	0.96	1.00	0.92	1.21	0.96	1.17
**May**	0.97	1.03	0.97	1.16	0.97	1.19
**Jun.**	0.98	0.99	0.97	1.14	0.97	1.20
**Jul.**	0.98	0.98	0.98	0.99	0.97	1.18
**Aug.**	0.98	0.92	0.97	1.09	0.97	1.15
**Sep.**	0.98	0.78	0.97	1.04	0.97	1.08
**Oct.**	0.98	0.74	0.96	1.22	0.97	1.04
**Nov.**	0.97	0.90	0.95	1.44	0.97	1.10
**Dec.**	0.97	1.02	0.95	1.32	0.96	1.08

**Table 4:** Root Mean Square Error (RMSE) values for air temperature vs. surface temperature in the central months of each season.

Month	Extent (°C)	< 2°C (%)	2–4°C (%)	4–6°C (%)	> 6°C (%)
**Jan.**	0.43–12.14	44.05	44.43	9.40	2.12
**Apr.**	0.45–14.70	19.02	31.21	35.22	14.55
**Jul.**	0.43–13.51	31.97	27.46	22.33	18.23
**Oct.**	0.40–13.05	20.31	48.75	24.96	5.97

**Fig 5:**
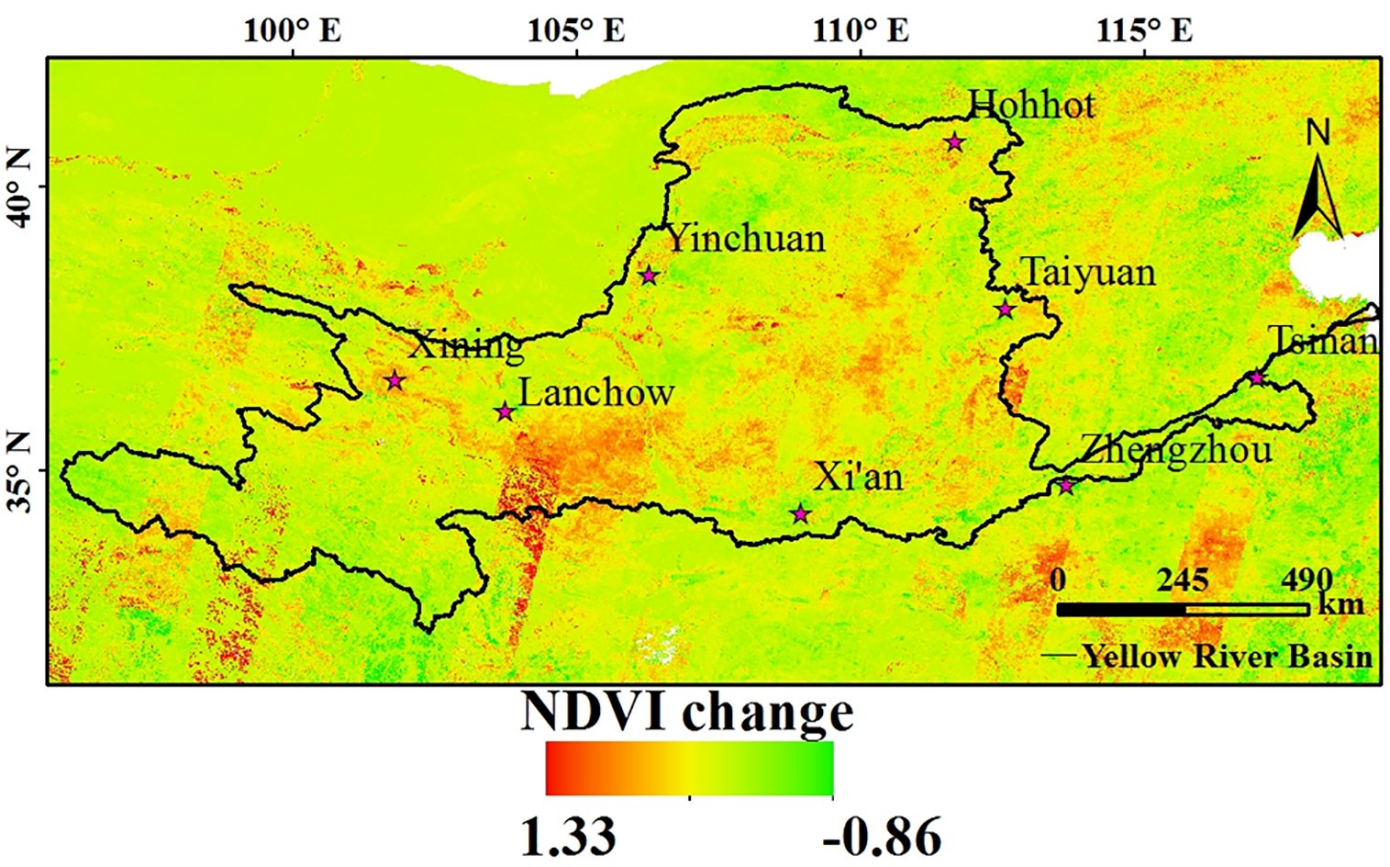
Spatial distribution of the NDVI change for the Yellow River Basin within the 2000–2015 period.

This analysis was performed for three reasons. First, the complex and diverse topographical conditions that characterise the Yellow River Basin (e.g., varying altitude and surface roughness and undulation) have a strong influence on air temperature. Second, the impact of vegetation cover on air temperature needs to be considered (Fig 5), i.e., the replenishment of vegetation in the growing season has a significant impact on surface temperature [49, 50], which could account for the significant difference between Ts and Ta during the growth period in the Yellow River Basin. Third, the complex geography, climate, ecology, and natural environment in the basin influence the inversion accuracy of the surface temperature to some extent, thereby enhancing the error associated with temperature estimates.

### Air temperature estimation and mountain-mass effect analysis

#### Air temperature estimation and precision evaluation

**Fig 6:**
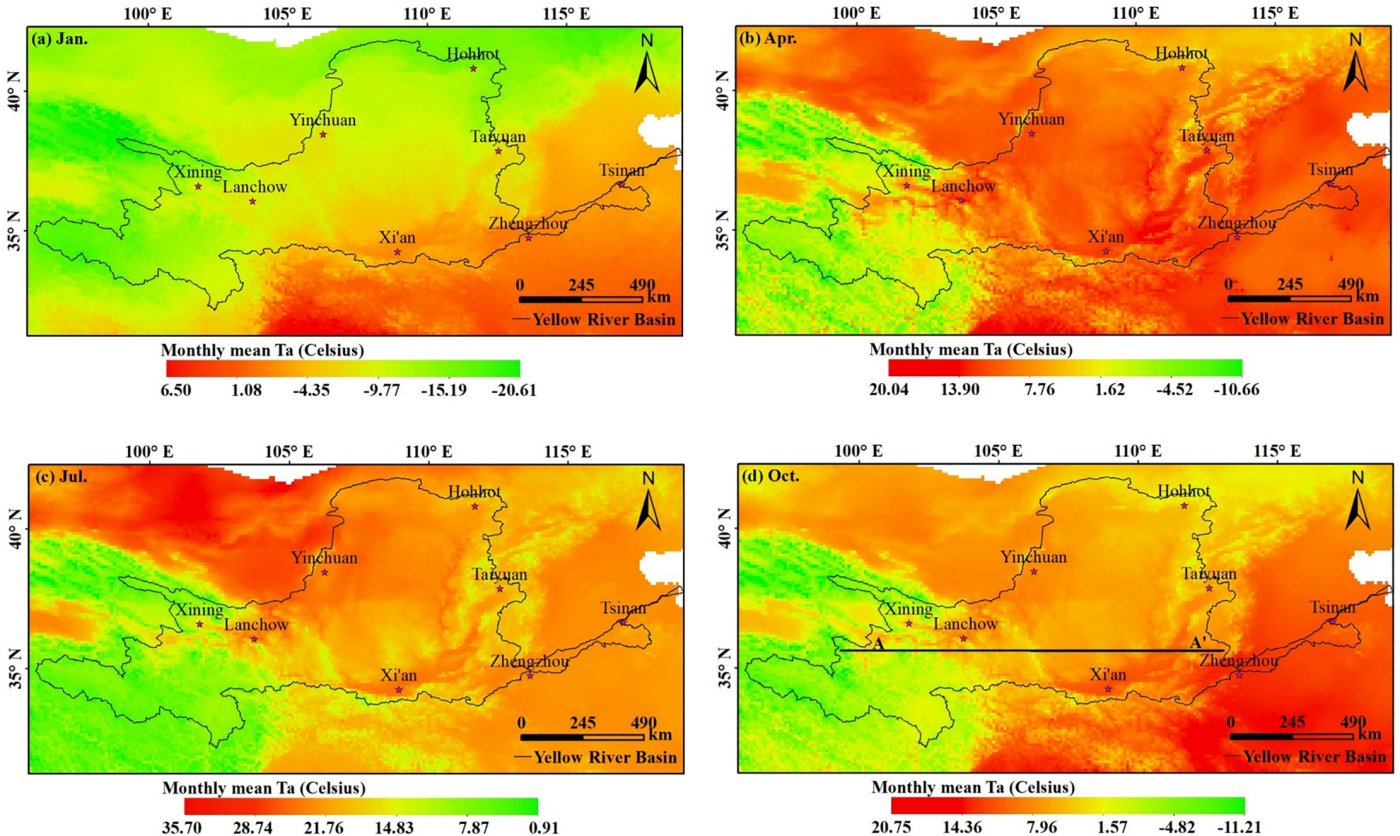
Spatial distribution of mean air temperature (Ta) values for (a) Winter, (b) Spring, (c) Summer, and (d) Autumn.

Table 5 shows that the 12-month decision coefficient (adjusted R^2^) was greater than 0.95 (0.96–0.98) and the RMSE was between 0.74°C and 1.03°C for each month. This is indicative of high accuracy. Further, the error verification results (Table 6) showed that, except for the highest errors, i.e., 1.04–1.11°C, which were observed from April to August, the estimation error was less than 1°C. The results also showed that the estimation accuracy of the GWR method could be controlled to within an error of 1.2°C. Furthermore, the accuracy of temperature estimates in the Yellow River Basin based on MODIS land surface temperature data, NMICD meteorological data, and DEM data was very high. The spatial distribution of estimated Ta showed that January was the coldest month, with a Ta between -20.61°C and 6.50°C (Fig 6A), whereas July was the hottest month, with a Ta between 0.91°C and 35.70°C (Fig 6C). Further, the air temperatures corresponding to each month were generally low in the west and high in the east; this is attributed to high-elevation plateaus and mountains in the west. The upper Tibetan Plateau showed low annual air temperatures with a narrow spatial distribution. However, air temperatures were high in the north-eastern area, upstream of the Inner Mongolia Plateau, owing to the rapid drying temperature rise close to the desert. Additionally, changes in the average monthly air temperatures in the middle and lower reaches of the Yellow River Basin were relatively high with a narrow spatial distribution; this is closely linked to the fact that these areas are located at low altitudes in the basin plain that continuously receive light and heat (Fig 6).

**Table 5:** Results of geographically weighted regression analysis for the central months of each season (R2: Coefficient of determination; RMSE: Root Mean Square Error).

Statistical index	Jan.	Apr.	Jul.	Oct.
**Sigma**	0.81	1.09	1.01	0.83
**R2**	0.98	0.96	0.98	0.98
**Adjusted R2**	0.98	0.96	0.98	0.98
**RMSE (°C)**	0.76	1.00	0.98	0.74

**Table 6:** Site verification error for the central months of each season (R2: Coefficient of determination; RMSE: Root Mean Square Error).

Month	Residual		Local R2	RMSE (°C)
	Range (°C)	-1.2–1.2°C (%)		
**Jan.**	-3.19–1.33	92.45	0.003–0.87	0.63–0.80
**Apr.**	-2.74–4.57	74.11	0.001–0.95	0.91–1.07
**Jul.**	-5.91–4.00	66.22	0.001–0.96	0.87–1.07
**Oct.**	-2.16–2.54	83.56	0.003–0.94	0.70–0.82

The annual average temperature in the Yellow River Basin was generally high in the south and east but low in the north and west. Additionally, the upper reaches exhibited the lowest temperatures in the entire basin. Moreover, the annual average temperature in the basin decreased with increasing latitude and elevation, and the lowest temperature in the basin was observed in January, during which time the Mongolian high pressure is particularly strong, and the temperature distribution varies with latitude. The temperature in the Inner Mongolia Plateau in the upper reaches of the basin was significantly low, making this region another low-temperature zone in the study area. Further, in the lower reaches of this river basin, the terrain is relatively flat and the mainstream flows in a north-eastward direction. The influence of cold air from the south-eastern parts of the East China Sea is greater in coastal areas than inland regions. Therefore, the temperature was high in the upstream region and low in the downstream region. Furthermore, owing to the effect of terrain and atmospheric circulation, the average temperature in January in the Yellow River Basin was lower than that in other parts of the world at the same latitude. This could be attributed to the fact that the Yellow River Basin is greatly affected by the East Asian winter monsoon; thus, January is the coldest month of the year in this basin. The temperature rose in February, reaching a maximum in July, before declining again in August as the summer monsoon weakened. Thus, the annual variation in temperature was steep and symmetrical, with spring temperatures slightly higher than those in autumn. Therefore, the relationship between terrain and temperature in the study area is highly complex.

#### Analysis of the mountain-mass effect in the Yellow River Basin

**Fig 7:**
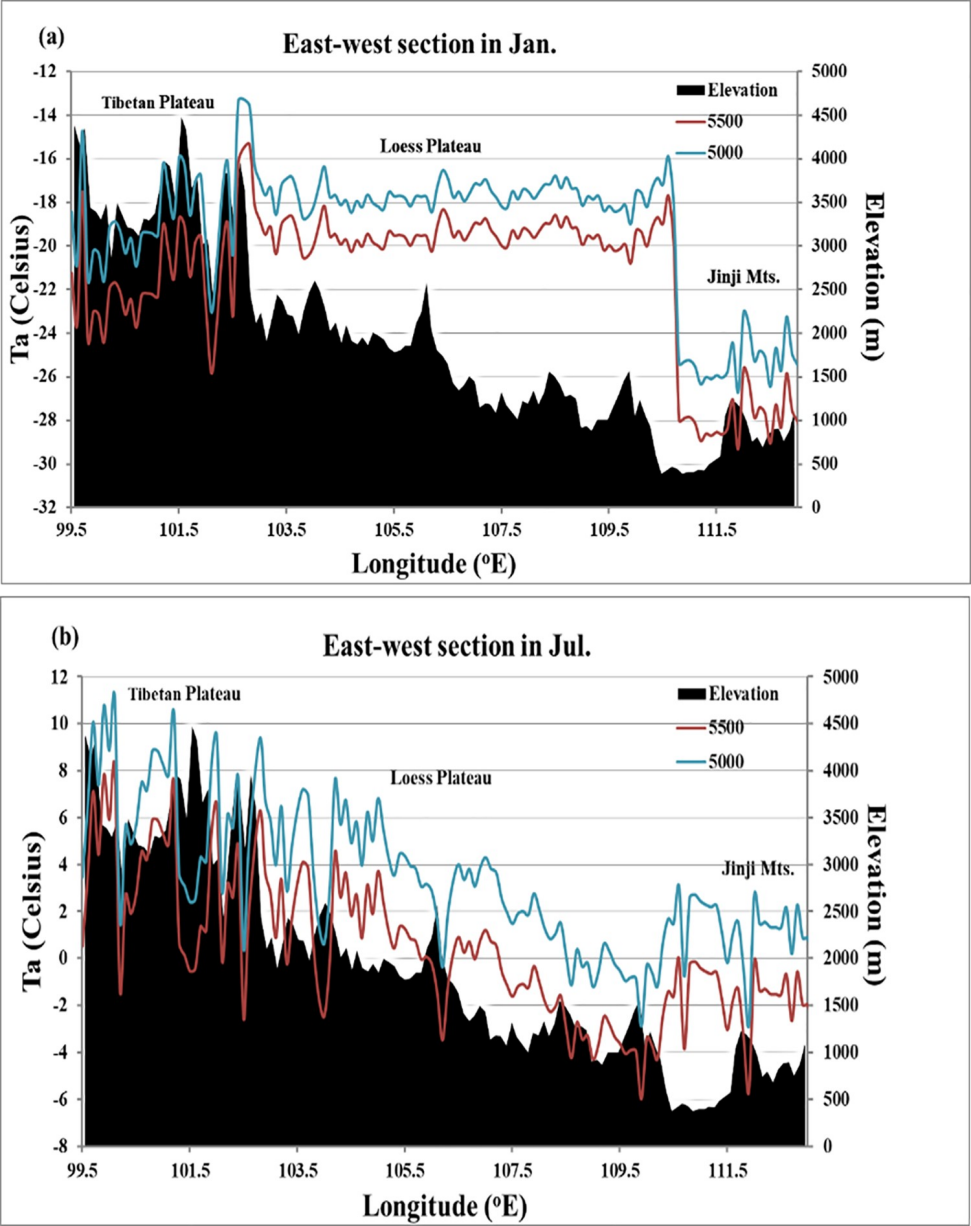
Temperature profile at 5,000 m and 5,500 m in the Yellow River Basin: (a) January; (b) July.

**Fig 8:**
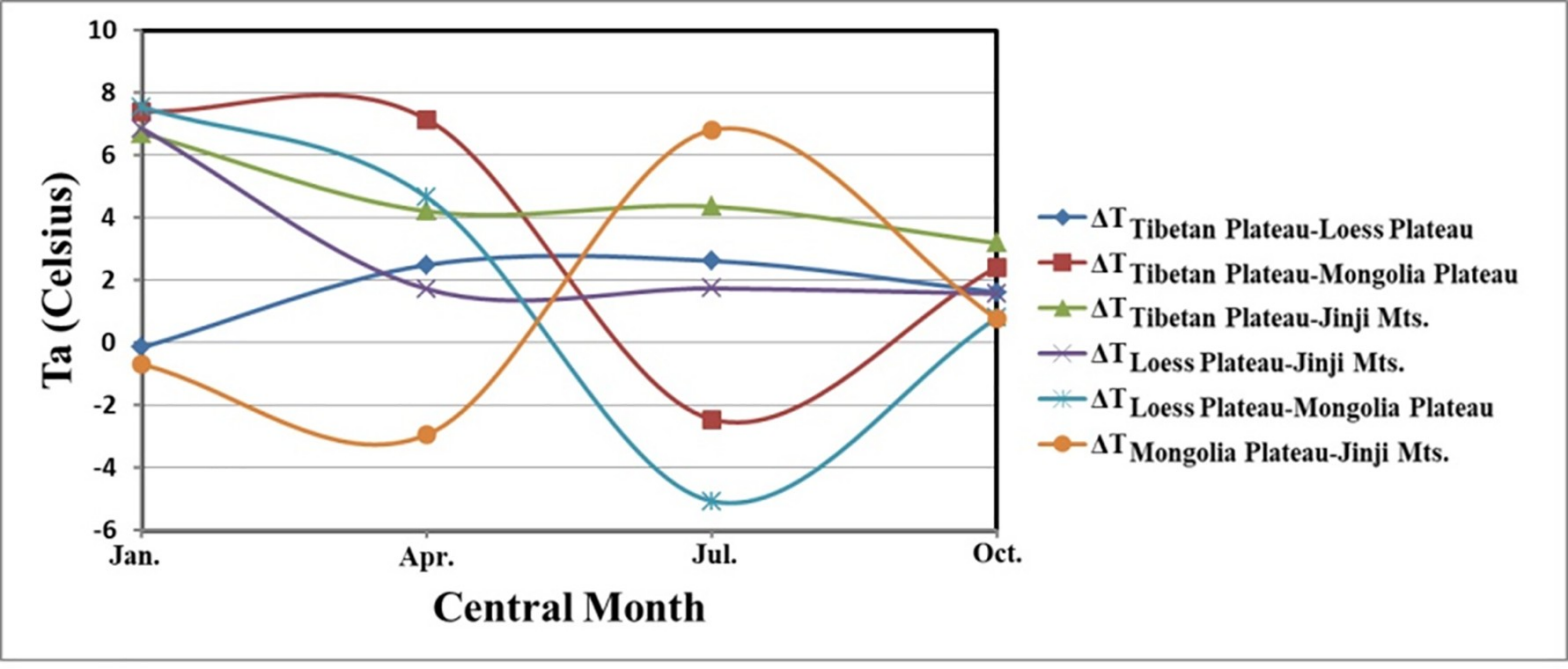
Temperature difference during the central months of each season at an altitude of 5,000 m.

To quantitatively analyse the mountain-mass effect in the Yellow River Basin, the air temperatures of different geomorphic units in the Yellow River Basin were compared at the same altitude, and the air-temperature differences were calculated. The average monthly air-temperature data corresponding to altitudes of 5,000 and 5,500 m were extracted in accordance with Eq (6) (Fig 7), and the air-temperature profile was created along the east–west direction at the same latitude (Fig 6(d)). Specifically, the air-temperature differences at an altitude of 5,000 m are shown in Table 7 and Fig 8. The air-temperature differences between the Loess Plateau and Jinji Mountain in January and July were 6.86°C and 1.74°C, respectively, that between the Tibetan Plateau and the Loess Plateau in July was 2.62°C, and those between the Tibetan Plateau and Jinji Mountain in January and July were 6.71°C and 4.36°C, respectively.

**Table 7:** Air and surface temperature differences between regions of the Yellow River Basin at an altitude of 5,000 m in the central months of each season.

Area	Jan.	Apr.	Jul.	Oct.
**Tibetan Plateau**	-18.64	-2.17	6.01	-3.10
**Loess Plateau**	-18.49	-4.66	3.39	-4.72
**Mongolia Plateau**	-26.04	-9.32	8.46	-5.52
**Jinji Mountain**	-25.35	-6.38	1.65	-6.29
**ΔT**_**Tibetan Plateau-Loess Plateau**_	-0.15	2.49	2.62	1.62
**ΔT**_**Tibetan Plateau-Mongolia Plateau**_	7.40	7.15	-2.45	2.42
**ΔT**_**Tibetan Plateau-Jinji Mts.**_	6.71	4.21	4.36	3.19
**ΔT**_**Loess Plateau-Jinji Mts.**_	6.86	1.72	1.74	1.57
**ΔT**_**Loess Plateau-Mongolia Plateau**_	7.55	4.66	-5.07	0.80
**ΔT**_**Mongolia Plateau-Jinji Mts.**_	-0.69	-2.94	6.81	0.77

However, owing to the influence of the temperature lapse rate, the temperature in the Loess Plateau in January was 0.15°C higher than that in the Tibetan Plateau in the same month. Additionally, the temperature of the Inner Mongolia Plateau in July was 2.45°C higher than that of the Tibetan Plateau, and 5.07°C higher than that of the Loess Plateau. Further, the temperatures of the Inner Mongolia Plateau in January and April were 0.69°C and 2.94°C higher than those in Jinji Mountain, respectively. Therefore, the temperature lapse rate has an important impact on the mountain-mass effect. Even though the temperature lapse rate varied seasonally, higher terrains showed more prominent mountain-mass effects at the same latitude. Thus, in the study area, the most prominent mountain-mass effect was observed in the Tibetan Plateau, followed by the Loess Plateau. For the central month of each season, at a given altitude, the temperature of the Tibetan Plateau was approximately 0–3°C higher than that of the Loess Plateau and approximately 3–7°C higher than that of Jinji Mountain.

Moreover, owing to the influence of the temperature lapse rate, the mountain-mass effect differed from one region to another. This study analysed the temperatures at altitudes of 5,000 and 5,500 m and the temperature differences throughout the basin, which revealed that the macroscopic influence of large terrain on temperature distributions and changes can be significant over a wide range, and the local topography can result in major temperature differences within a short distance. Comprehensive and accurate data on the factors affecting air temperature (e.g., soil temperature and humidity, vegetation cover, mountain-mass effect, slope topographic conditions, and precipitation) at the corresponding scales should be obtained in future studies to improve our estimates of the mountain-mass effect.

## Discussion

### Characteristics and causes of the vertical temperature gradient

Owing to the scarcity of meteorological stations in the interior parts of the plateaus and large mountain ranges, it is difficult to accurately express the rate of vertical temperature decline in such areas. Particularly, the impact of the mountain-mass effect on the temperature of large plateaus and mountains hinders an accurate determination of the rate of vertical temperature decline [51–54]. This implies that, to accurately estimate the temperature-increasing effect according to the temperature values of different pressure surfaces from the sounding data, it is helpful to show the complexity and variability of the vertical decline rate of air temperature in different regions [55–58]. Therefore, in this study, we calculated the vertical decline rate of temperature in each month based on the 16-year monthly average temperature of the eight pressure surfaces (1,000, 925, 850, 700, 600, 500, 400, 300, 250 hPa) of NCEP and ECMWF data (Table 8) [59]. As the temperature value corresponding to each air pressure surface represents the average temperature of a grid square with a horizontal resolution of 2.5 × 2.5, superposing these nine isobaric surfaces can reveal the temperature value of the eight air pressure surfaces in each grid. However, not all areas have all eight air pressure surfaces. In high-altitude plateaus, such as the Tibet Plateau, the initial air pressure on the ground may be less than 600 hPa [60]. Therefore, to accurately reflect the actual atmospheric temperature distribution, the pressure-surface temperature data and the DEM were superimposed to remove the temperature of the pressure surface estimated below the surface, retaining only the temperature data corresponding to the pressure surface above the ground (Eq 13). From 500 to 100 hPa, a gradual decrease in temperature was observed with height. Further, the cold source characteristics in winter were more obvious in the Tibet Plateau, and the cooling trend of the Yellow River Basin gradually weakened from west to east. As the degree of change of γT with height differed, the values between the layers were also significantly different. Thus, the overall distribution of γT in the middle and lower troposphere of the Yellow River Basin was high in the west and low in the east, north, and south, with the value of γT increasing with height. Moreover, the distribution of isothermal gradients was densest at the junction of the Tibet Plateau and Loess Plateau. In summer, the γT on the Tibetan Plateau showed the most drastic changes, with the most significant variation with height observed in winter. Additionally, γT appeared weaker in summer than in winter at low latitudes, but stronger in summer than in winter at mid–high latitudes. The troposphere in the Loess Plateau, Mongolia Plateau, and Jinji Mountain is larger than that in the Tibetan Plateau. This indicates that the troposphere temperature in non-plateau areas is not affected by the plateau topography. It was also observed that the degree of decrease in γT with height was greater in non-plateau areas than in the plateau area. Furthermore, in winter and spring, changes in γT were more susceptible to cold air or other factors, and the overall trend of T in non-plateau regions was weaker and less significant than that in plateau regions. The vertical change in the temperature gradient over the plateau area showed very obvious seasonality.

**Table 8:** Lapse rates for the mean air temperature of different reanalysis data at the regional scale (°C/100 m).

Area	ECWMF				NCEP			
	Spring	Summer	Autumn	Winter	Spring	Summer	Autumn	Winter
**Tibetan Plateau**	-0.36*	-0.35*	-0.35*	-0.36*	0.04	0.04*	0.03	0.02*
**Loess Plateau**	-0.41*	-0.44*	-0.31*	-0.30*	-0.17*	-0.16*	-0.13*	-0.14*
**Mongolia Plateau**	-0.36*	-0.39*	-0.31*	-0.30*	0.07	0.02	0.09*	0.18*
**Jinji Mountain**	-0.27*	-0.29*	-0.22*	-0.16*	-0.01	-0.02*	-0.01	-0.02

* denotes the 95% confidence level.

### Temperature lapse rate in the Yellow River Basin

The rate of vertical temperature decline is key to determining the geographical distribution of temperature, and is an important measure for predicting the height distribution of vegetation via the application of bioclimatic indicators. It also plays an important role in many fields, including forest management, agriculture, and ecology). In previous studies, fixed vertical temperature decline rates, e.g., 0.6°C/100 m and 0.61°C/100 m, were used to establish a daily temperature grid [61], calculate the climate indicators related to the upper and lower limits of a species, and analyse the eco climatic characteristics of the forest line [40]. However, due to the influence of macro factors, such as altitude, latitude, and sea-land distribution, as well as local factors, such as inversion and cold lakes, determining the spatial distribution of the vertical temperature decline rate is extremely complicated. From coastal to inland regions, the vertical temperature decline rate in January and July, as well as the annual average in China, first decreases, then increases, then decreases gradually [62].

If a fixed vertical decline rate is used to estimate the temperature in different regions, a large error may be generated. For example, in Sichuan, which is located in Leshan City, at an altitude of 424 m, the hottest monthly average temperature is 25.9°C (GHCN, average value corresponds to the 1950–2000 period). Moreover, based on a vertical temperature decline rate of 0.6°C/100 m, it is estimated that the temperature of the hottest month in Mount Emei (3,049 m a.s.l.), which is only 37 km from Leshan City, is 10.15°C. However, the actual average temperature during the hottest month at this mountain is 12.31°C (GHCN, the average value in 1951–2000). Furthermore, the temperature of the hottest month in Linzhi (3,000 m a.s.l.) is 16.7°C (~1950–2000, annual average). Based on a vertical temperature decline rate of 0.6°C/100 m, the estimated temperature of Nyingchi Sejila Mountain Forest Line (4,300 m a.s.l.) is 8.9°C, whereas the hottest monthly temperature at the forest line according to the records of Sejila Mountain Meteorological Station is 9.4°C [63].

The near-surface-temperature lapse rate based on data from surface weather stations can provide a certain reference for the spatial interpolation of temperature. However, in the western mountainous area, especially the Tibetan Plateau, the meteorological stations are sparsely distributed, and the elevation is not representative. Thus, the temperature lapse rate in different elevation ranges may differ significantly. Therefore, extra care should be taken when using the temperature lapse rate.

Additionally, the difference in slope aspect and the mountain-mass effect can also cause differences in the rate of temperature decline. The influence of slope aspect and mountain-mass effect can be further considered on the basis of comprehensive natural zoning.

### Limitations of the study

The mountain-mass effect is a thermal effect caused by the higher elevation of the ground surface in mountainous regions than in the surrounding lowlands, which leads to higher temperatures and higher vertical belt boundaries in mountainous areas. Therefore, the temperature difference between the interior and exterior of mountain ranges should be an ideal indicator of the magnitude of the mountain-mass effect. However, many factors affect this temperature difference, including atmospheric and geographic factors of various scales.

It is implied that the height of the ground surface is the root cause of the mountain-mass effect, the temperature difference is the climatological mechanism, and the vertical zone height difference determines the performance of vegetation. Moreover, dry and wet climates in mountainous areas affect the temperature and height of the vegetation distribution. In other words, temperature differences between mountainous and non-mountainous areas at the same altitude not only depend on the height of the ground surface, but also on the general climate. Thus, the mountain-mass effect is not strictly equivalent to the temperature difference as it also depends on several other factors.

Further, although the temperature difference appears to have the greatest influence on the mountain-mass effect, there are several uncertainties related to calculation of the temperature difference. For example, the temperature difference between a certain point in a mountainous area and the free atmosphere at the same altitude in a non-mountainous area can only be estimated using the vertical decline rate. Moreover, the temperature values corresponding to mountainous areas have considerable errors owing to the scarcity and limited access of meteorological stations. Furthermore, the height difference of vertical zones based on the temperature difference adds more uncertainty due to differences in ground material, terrain slope, plant species, etc.

Although this study focused on the estimation of mean values, the method proposed herein can also be applied to determine daily maximum, minimum, and average temperatures. Additionally, more variables (e.g., NDVI, precipitation, and albedo) should be considered in future research to explore their effect on the accuracy of model estimation.

## Conclusions

Time series and regression analyses of MODIS land surface-temperature and air-temperature data showed similar temporal variations for these two temperatures and a good correlation between these data. Therefore, MODIS surface-temperature data can be used to estimation the temperature in mountainous areas. The results of the geographically weighted regression analysis with MODIS surface temperature and altitude as independent variables showed that the GWR method, combined with meteorological data, MODIS surface-temperature data, and DEM data, allows the accurate estimation of air temperature in the Yellow River Basin. We also compared NMICD, CRU, and APHRODITE data. Considering the resolution of these three different data products and the results of their accuracy estimation tests, NMICD data were selected in this study. Although CRU and APHRODITE data were still highly effective for surface temperature estimation, these datasets exhibited lower accuracy. Additionally, the downscaling method was employed to improve the applicability of these two types of data.

Temperature estimates in the Yellow River Basin over 12 months were used to obtain the temporal variation of temperature in the study area. It was observed that the temperature lapse rate played an important role in estimating the mountain-mass effect, which could not be accurately estimated based on a fixed temperature lapse rate of the average temperature. Furthermore, the spatial distribution pattern of the temperature lapse rate and the mountain-mass effect was used to effectively express the change in temperature trends for plateaus, mountains, basins, and other geomorphic areas that are greatly affected by mountains. Generally, at the same altitude and latitude, the Tibetan Plateau had a higher temperature than the Loess Plateau and Jinji Mountain. However, these regions exhibited different sensitivities of air temperature and land-surface temperature to local and surface energy; thus, the temperature lapse rate was unstable. This implies that the constant environmental lapse rate typically assumed in previous studies (0.65°C/100 m) is inappropriate for the Yellow River Basin. In summary, we employed meteorological data and corresponding MODIS surface-temperature data, regression analysis of DEM data, and verification of the results using meteorological station-based data to estimate relatively accurate temperatures for the Yellow River Basin and determine regional differences in the mountain-mass effects. This research provides a foundation for quantifying the ecological effects of mountainous plateaus.
